# Confirmation of a measurement model for hospital supply chain resilience

**DOI:** 10.3389/fpubh.2024.1369391

**Published:** 2024-05-22

**Authors:** Baoyang Ding, Xiaohan Yang, Tiantian Gao, Zheng Liu, Qiang Sun

**Affiliations:** ^1^Centre for Health Management and Policy Research, School of Public Health, Cheeloo College of Medicine, Shandong University, Jinan, Shandong, China; ^2^NHC Key Lab of Health Economics and Policy Research (Shandong University), Jinan, Shandong, China; ^3^Department of Pharmacy, Shandong Provincial Hospital Affiliated to Shandong First Medical University, Jinan, Shandong, China

**Keywords:** supply chain resilience, hospital supply chain, scale development, dynamic capability theory, China

## Abstract

**Background:**

The hospital supply chain has revealed increasing vulnerabilities and disruptions in the wake of the COVID-19 pandemic, threatening the healthcare services and patient safety. The resilience of hospital supply chains has emerged as a paramount concern within the healthcare system. However, there is a lack of systematic research to develop an instrument tailored to the healthcare industry that is both valid and reliable for measuring hospital supply chain resilience. Therefore, this study aims to construct and validate a comprehensive scale for assessing hospital supply chain resilience, based on dynamic capability theory.

**Methods:**

This study followed rigorous scale development steps, starting with a literature review and 15 semi-structured interviews to generate initial items. These items were then refined through expert panel feedback and three rounds of Delphi studies. Using data from 387 hospitals in Province S, mainland China, the scale underwent rigorous testing and validation using structural equation modeling. To ensure the most effective model, five alternative models were examined to determine the most suitable parsimonious model.

**Results:**

The study produced a 26-item scale that captures five dimensions of resilience in line with dynamic capability theory: anticipation, adaptation, response, recovery, and learning, all showing satisfactory consistency, reliability and validity.

**Conclusion:**

The multi-dimensional scale offers hospital managers a valuable tool to identify areas needing attention and improvement, benchmark resilience against their counterparts, and ultimately strengthen their supply chains against unexpected risks.

## 1 Introduction

Hospital supply chain faces increasing vulnerability due to a range of disruptive events, including sudden shortages of raw materials, production halts, and unforeseen public health crises (1, 2). These disruptions not only jeopardize the stability and security of the supply chain to provide the medication and equipment used by patients, but also pose significant risks to the quality of healthcare services and patient safety (3). As a strategic approach to mitigate the adverse effects of disruptions, the resilience of supply chain become heightened in recent years, particularly in the wake of global disruptions such as the COVID-19 pandemic (4, 5).

The pursuit of enhancing resilience within hospitals necessitates a thorough examination of their vulnerabilities and the identification of weaknesses in their resilience capabilities. Despite the critical importance of developing resilience to supply chain disruptions, research in this area remains scant. While existing literature has introduced several measurement scales of supply chain resilience (6–9), these are primarily tailored to the upstream supply chains of manufacturing enterprises, neglecting the unique characteristics of hospital supply chains. Distinguished by the specialized demands of the healthcare industry, hospital supply chains must be prepared for extraordinary events such as pandemics, chemical/biological threats, and widespread illness outbreaks (10). The complexities of hospital supply chains, the movement of highly valuable commodities, and the overarching imperative to protect human lives, justify tolerating certain inefficiencies, such as maintaining higher-than-usual stock levels, which would be deemed unacceptable in other contexts, particularly in non-profitable public hospitals (2, 11). Given the distinct challenges and the pivotal role of hospital supply chains in disaster response and routine healthcare delivery, there is an urgent need for a resilience assessment framework specifically designed for healthcare institutions. Such a framework should address their unique challenges and requirements, rather than merely replicating approaches from other sectors.

In this context, Dynamic Capability Theory (DCT) offers a powerful lens through which to examine and enhance resilience. As articulated by Teece (12), DCT underscores the importance for organizations to sense and seize external opportunities and threats, and to integrate, build, and reconfigure internal and external competencies to navigate rapidly evolving environments. The dynamic nature of supply chain disruptions, as starkly illustrated by the COVID-19 crisis, calls for a theoretical approach capable of accommodating the unpredictable and fluid nature of these challenges in hospital supply chains (13). DCT, with its focus on the capabilities needed to adapt to changing conditions, mitigate vulnerabilities, and reconfigure resources to ensure the delivery of high-quality patient care (14), provides an apt framework for this endeavor. Thus, this study aims to develop a comprehensive assessment model to evaluate the resilience of hospital supply chains, drawing on the insights of dynamic capability theory.

## 2 Literature review

Resilience, as a multidisciplinary concept, has become increasingly vital in the supply chain field, especially after the 9/11 attacks in the United States exposed the intrinsic fragility of supply chain networks. Scholars such as Rice and Caniato (15), and Christopher and Peck (16), were pioneers in defining supply chain resilience (SCRE) as an organization’s ability to respond to unforeseen disruptions and restore to normal operations. Despite these foundational efforts, the academic discourse continues to debate with how to precisely conceptualize and measure resilience, highlighting a gap that particularly affects the specificity required for hospital supply chains (17).

In addressing this gap, the integration of DCT with resilience concepts, underpinned by operations management strategies, offers a promising path for examining pandemic impacts on supply chains (18). Originating from the Resource-Based View, which focuses on an organization’s internal resources, DCT expands this view by also considering how an organization’s capabilities can be adapted or transformed in response to external environmental shifts (9). This external focus is particularly relevant for hospital supply chains, which are embedded in a complex network of stakeholders including suppliers, healthcare providers, and patients, and are subject to a myriad of external influences.

The literature on supply chain resilience and DCT can be divided into distinct perspectives, and yielding divergent measurement approaches (19). One perspective categorizes resilience into temporal phases: pre-disruption, during-disruption, and post-disruption, correlating with proactive, concurrent, and reactive capabilities, respectively. Proactive capabilities involve anticipation, preparation, and planning (8, 20), while concurrent capabilities focus on adaptation, response, and coping during disruptions (21). Reactive capabilities are centered on recovery and bouncing back post-disruption (22). Notably, concurrent strategies are occasionally seen as a subset of reactive strategies due to their immediate nature in addressing disruptions, as evidenced in the work of Chowdhury and Quaddas (9).

An alternative perspective scrutinizes supply chain resilience through the lens of essential capabilities, which have notably evolved from a primary emphasis on reactive responses and recovery to a broader, more inclusive focus (15, 16). This expanded view encompasses the abilities to anticipate, adapt, respond, recover, and learn from unexpected events, disruptions, and the aftermath of such incidents (4, 19, 23). Despite this progression, much of the existing literature still adheres to narrower definitions of supply chain resilience, often selecting specific resilience capabilities for measurement, with a predominant focus on response, recovery, and adaptation. In contrast, Ali et al. (19) have put forward a more comprehensive framework that encapsulates all pivotal capabilities, including the ability to anticipate, adapt, respond, recover, and notably, the ability to learn from experiences and failures. The findings reinforce the argument that supply chain resilience research should expand its focus to encompass a broader spectrum of proactive capabilities, as well as the capacity for learning from past experiences to enhance resilience. Considering a pressing need for the development of more nuanced constructs for measuring hospital supply chain resilience, this study pursues to construct a more comprehensive and effective framework based on dynamic capability theory.

## 3 Methods

**Figure 1:**
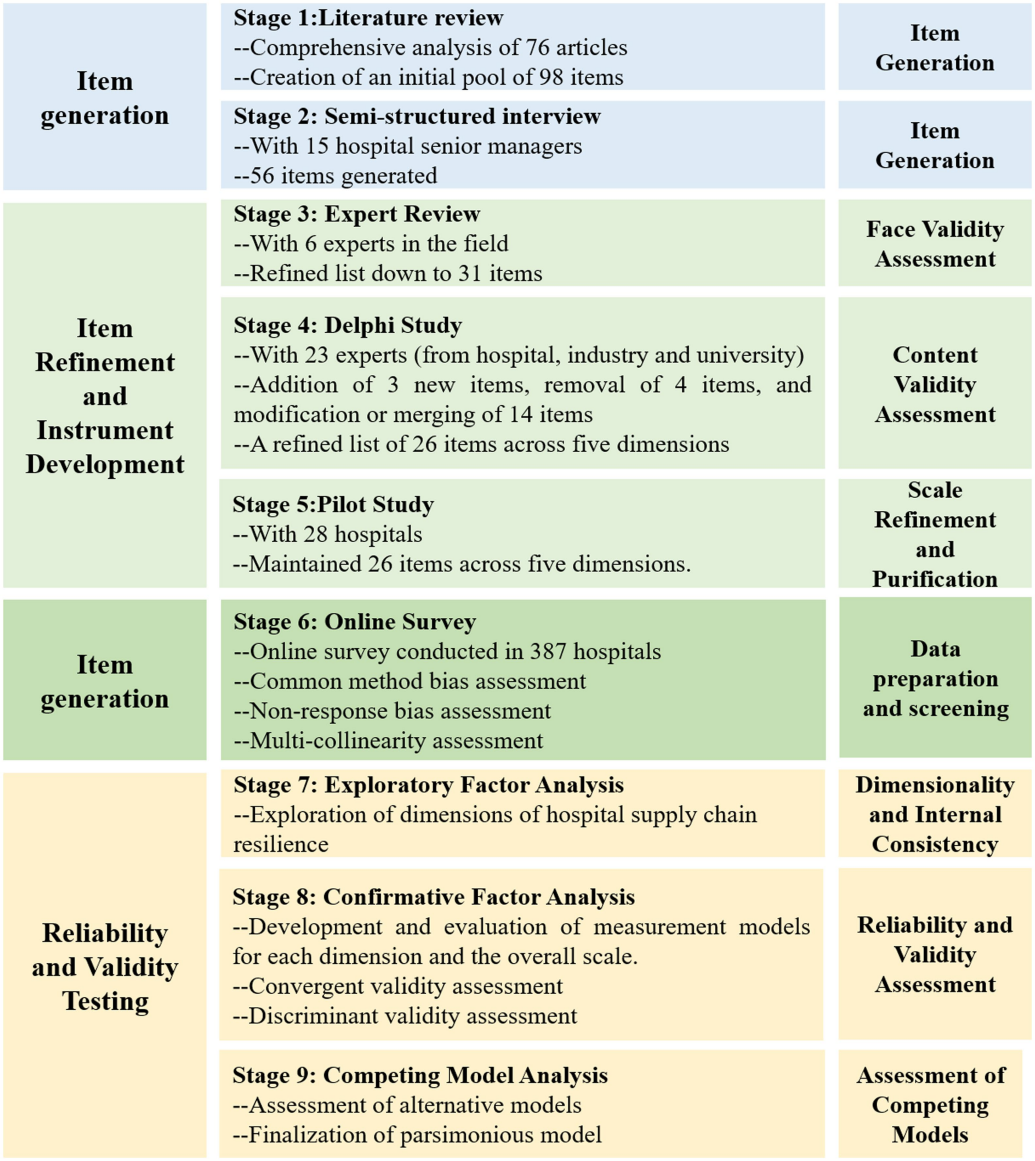
Scale Development Process.

This study adheres to a widely recognized scale development procedure (24, 25), as outlined in Figure 1. The process commences with generating initial measurement items from an extensive literature review and semi-structured interviews with domain experts. These items are then refined through an expert review process and three rounds of Delphi studies, aimed at achieving consensus and ensuring content validity. A pilot survey further tests the practical applicability of the refined items. The reliability and validity of the final scale are rigorously assessed through exploratory factor analysis and confirmatory factor analysis, alongside competing model analysis to confirm the scale’s structural integrity. This multi-stage process combines theoretical insights with empirical validation, ensuring the development of a robust, reliable, and applicable measurement scale for assessing resilience in hospital supply chains. The following sections provide detailed descriptions of each stage.

### 3.1 Initial item generation

#### 3.1.1 Literature review

A comprehensive literature review was conducted using a set of keywords such as “hospital supply chain,” “healthcare supply chain,” “resilience,” “measurement,” and “scale.” This preliminary search encompassed six databases: Web of Science, Science Direct, Emerald, Scopus, Wiley Online, and PubMed. From this effort, 76 articles were selected for an in-depth review. This study conducted a collaborative and deductive coding process by two researchers in line with the framework suggested by Ali et al. (19), in which the five main dimensions of supply chain resilience were illustrated from dynamic capability perspective. It summarized the five key dimensions comprising of ability to anticipate, ability to adapt, ability to response, ability to recover and ability to learn. An initial list of 98 items across these five dimensions of supply chain resilience was established.

#### 3.1.2 Interviews with experts

Given the scarcity of literature specifically addressing the healthcare industry, and more precisely, the downstream context of hospital supply chains, this study engaged in semi-structured interviews with 15 hospital supply chain senior managers. To systematically manage and analyze the qualitative data gathered from these experts, this study utilized NVivo 12 software to perform deductive coding procedures efficiently. After cross-validation by two researchers, the thematic coding process ultimately revealed a total of 56 items that underscore the five dimensions of resilience. The details were shown as following Table 1.

**Table 1:** Items generated from expert review.

Capability	Code Content (Frequency)
Anticipate	Information Transfer (12), Advance Ordering (12), Demand Forecasting (10), Training (9), Forecasting by Experience (8), Safety Awareness (8), Mastering Information (7), Understanding Risks in Advance (5), Advance Communication (5), Constant Communication (5), Information Lag (5), Proactive Communication (4), Supplier Out-of-Stock Notification (3), Preparing Contingency Strategies in Advance (3), Risk Awareness (2), Safety Training (2), System Alarms (2), Establishing Contacts (2), Strengthening Learning (2)
Adapt	Safety Stock (15), Advance Stocking (14), Multiple Suppliers (13), Dual Sourcing for Single Product (12), Stockpiling Special Items (8), Emergency Ordering (8), Emergency Delivery (7), Staff Rotation (7), Multiple Sourcing for Single Product (7), Mentoring by Experienced Staff (6), Knowledge Transfer (5), Categorizing Products (5), Finding Substitutes (4), Reserve Equipment (2)
Respond	Feedback Information (10), Setting Responsible Personnel (8), Information Exchange (8), Supply Coordination (7), Communication with Departments (5), Efficiency Improvement (5), Emergency Plans (5), Feedback System (4), Setting Up Substitutes in Advance (4), Emergency Team (3), Coordinating Command (3), Time Saving (2), Joint Response (2)
Recover	Prioritize resources on critically demand (11), Product coordination (8), Reducing impact (6), Communication with Suppliers (6), Management Measures (5), Employee Enthusiasm (5), Reallocating Resources (5), Improving Administrative Efficiency (4), Re-bidding (4), Top-Down Communication (3), Process Adjustment (2), Seeking Help and Support (2)
Learn	Learning from Other Hospitals’ Experiences (13), Establishing Trust with Multiple Parties (9), Summarizing Experience (8), Targeted Training (7), Continuous Learning (5), Continuous Improvement (4), Introducing New Technologies (4), Innovative Methods (3), Monitoring Performance Changes (2), Increasing Partnerships (2)

#### 3.1.3 Panel review

The initial list of items, derived from both the literature review and expert interviews, underwent careful evaluation by another panel of experts, which included two senior hospital managers, two scholars specializing in hospital supply chains, and two industry experts. Ambiguous item definitions were clarified to better align with the context of hospital supply chains, and items that were redundant were either eliminated or combined with similar ones. As a result, 31 items with robust face validity were retained for subsequent analysis.

### 3.2 Item review and sorting by Delphi study

To ensure the content validity of the study, the Delphi method was employed, leveraging its structured approach to facilitate consensus among a panel of experts through an iterative process. This method was instrumental in refining the conceptual framework for the hospital supply chain resilience assessment scale. Utilizing a preliminary assessment system, a bespoke expert consultation questionnaire was developed, adhering to a Likert five-point scale. This allowed experts to evaluate the necessity of each item, details of which are provided in a supplementary document. A total of 23 experts participated in all three rounds of the study. The composition of the sample is detailed in supplementary document. The SurveyStar Platform, an online survey tool, was utilized for the distribution and collection of the expert consultation forms, with each expert individually completing the questionnaire. Following each consultation round, the collected expert opinions were subjected to statistical analysis. Through discussion, indicators deemed unnecessary were eliminated, and new indicators were identified for inclusion. The results of expert opinions on each indicator were then fed back to the participants in the subsequent round of consultation, ensuring a transparent and iterative refinement process.

The experts’ familiarity with the subject matter, as indicated by the Cs score, was 0.820, while the basis of their judgments, as indicated by the Ca score, was 0.904. The expert authority coefficient (Cr) was calculated to be 0.862. In each expert consultation round, 23 questionnaires were distributed and 23 valid questionnaires were collected, maintaining a 100% response rate. The first round elicited feedback from 13 experts, and the second round from 3 experts, with feedback rates of 57 and 13%, respectively. The Kendall’s coefficient of concordance (W) during the three rounds of expert consultation was statistically significant and stable between 0.5–0.6 (shown as Table 2), demonstrating good consensus and high credibility achieved through this iterative expert consultation process in the process and no further rounds needed.

**Table 2:** Results of Kendall’s coefficient of concordance (W).

Round	Statistical Tests	Indicator Results	
		First-order indicators	Second-order indicators
Round 1	W	0.273	0.314
	*χ^2^*	25.145	231.216
	*df*	4	30
	*P*	<0.001	<0.001
Round 2	W	0.535	0.511
	*χ^2^*	49.255	352.319
	*df*	4	27
	*P*	<0.001	<0.001
Round 3	W	0.584	0.545
	*χ^2^*	53.764	25
	*df*	4	351.183
	*P*	<0.001	<0.001

Following three rounds of Delphi studies, three new indicators were added, 4 secondary indicators were removed, and 14 secondary indicators were either modified or merged. This led to the establishment of a hospital supply chain resilience assessment scale comprising 5 primary indicators and 26 secondary indicators. The details of each item were shown in Table 3.

**Table 3:** Measurement scale used for main survey.

Item	Measurement items
Anticipate Capability	
Mapping Vulnerability (8, 26–29)	We proactively identify potential risk factors in our supply chain that may lead to vulnerabilities and are quick to detect product stockouts.
Forecasting (7, 27, 30)	We can effectively forecast the demand for key products in use at the hospital to avoid potential risk events.
Supply Chain Robustness (31–33)	Even in the event of a product supply disruption, our hospital’s supply chain network and core suppliers can maintain stability over an extended period, as before the risk event.
Information Transparency (7, 8, 22, 27, 34, 35)	We can monitor the entire process from the supplier to clinical department in real-time through an integrated information system.
Risk sharing (7, 30)	We actively establish strategic relationships with key supply chain partners to control and share risks.
Risk Management Culture (17, 27, 29, 30, 33)	Our managers strive to incorporate potential risks into decision-making, and reinforce employees’ risk and safety management awareness.
Adaption Capability	
Supplier Flexibility (7, 27, 29, 34)	We have alternative products or multiple supply channels for our products.
Process Flexibility (27, 34, 36, 37)	In the face of uncertainty, we can flexibly adjust supply chain processes.
Institution Flexibility*	We have comprehensive product management and usage institutions that can be continuously improved in response to external policy adjustments and hospital demand changes.
Inventory Redundancy (7, 27, 29, 33, 38, 39)	We have set safety stock levels for our products.
Equipment Redundancy (27, 33, 39)	We possess various backup facilities and equipment for emergencies.
Personnel Redundancy	To mitigate potential staff shortages, we regularly rotate employees to adapt to various job positions.
Response Capability	
Collaborative Decision-Making (7, 27, 35, 40)	We plan and make supply chain decisions collaboratively with hospital suppliers, clinical departments, and other divisions to address risks.
Information Sharing (7, 17, 27, 29)	We actively share and communicate information related to risk events in real-time with key supply chain partners.
Responsiveness (27, 33)	We have a well-established and responsive risk information feedback mechanism, allowing management to understand supply chain risks promptly in both daily and risky situations.
Response Speed (9, 27, 33, 41)	We can respond quickly to disruptions, such as finding alternative products immediately.
Emergency Planning (26, 27, 29, 42)	We have an emergency response team and comprehensive emergency plans for handling supply chain risk situations.
Recovery Capability	
Resource reconfiguration (8, 23)	We have the ability to reallocate resources and resume normal supply chain operations quickly after a disruption.
Supply chain reconfiguration (42, 43)	When facing risk events, we can quickly adjust the supply chain structure and operational processes to adapt to changes and recover rapidly.
Efficiency (7, 33, 35)	Our employees are highly efficient and capable of promptly handling assigned tasks to ensure a rapid recovery following supply chain disruptions.
Government Support (7, 27, 28, 34)	The government provides us with ample support when we face supply chain risk events.
Learning Capability	
Post-Event Feedback (27, 39)	Following risk events like product stockouts, we consistently conduct reviews to learn from our experiences.
Post-Event Training and Education (7, 9, 27, 29, 44)	Based on past risk events, we provide targeted skill training for our employees to prepare for future risks.
Continuous Innovation (7, 33, 45)	We apply innovative methods and digital technologies to improve emergency plans and conduct post-event training.
Inter-Organizational Learning (27, 29, 33, 40, 46)	We maintain good communication and mutual learning with supply chain stakeholders to prepare for future risks.
Trust (17, 27, 33, 47)	We establish strong trust with key supply chain partners to jointly enhance emergency preparedness capabilities.

*: item added by Delphi study.

### 3.3 Pilot survey and questionnaire development

To ensure the suitability of primary questionnaire, a pilot survey was conducted on a small scale before the distribution of the large-scale formal questionnaire. Feedback regarding the format, descriptions, and logic of the questionnaire was sought to adjust the order of the questions, ensuring logical flow and coherence throughout. Employing purposive sampling, the study targeted the pharmaceutical and consumable management departments of public hospitals rated secondary level and above. A total of 30 pre-survey questionnaires were distributed, with 28 valid responses received. The reliability of the entire questionnaire was 0.941, confirming its suitability for data collection.

### 3.4 Sample and data collection

The final version of the questionnaire consisted of two parts: basic hospital information and the hospital supply chain resilience measurement scale (shown in supplementary document). The basic hospital information section included hospital location, type, level, supply chain operation mode, and other details, with question types being multiple-choice and fill-in-the-blank. The hospital supply chain resilience assessment scale comprised 5 dimensions with a total of 26 indicators, utilizing a 7-point Likert scale to minimize the role of ambiguity in the answers. The questionnaire was distributed through the SurveyStar Platform, currently the most popular online survey platform in Mainland China.

The sample collection for the questionnaire received support from the provincial health commission of S Province, and was distributed to all 617 public hospitals rated secondary level and above within the province. Excluding hospitals that had already participated in the preliminary survey phase, questionnaire links were sent to the remaining 589 healthcare institutions, in which the senior manager who is in charge of pharmaceutical products or medical consumable department answered the questionnaire. All questionnaires were collected anonymously within 2 weeks and a reminder message was sent by the end of the first week. In total. 392 responses ultimately retrieved. After removing hospitals with shorter response times and those with missing values, a final sample of 387 was retained for the ultimate reliability and validity analysis.

### 3.5 Data analysis

Given that the overall response rate for the study was 65.7% and that data collection spanned a two-week period, there is a possibility of late response bias. To address this, the study classified respondents into two groups: early respondents, who participated within the first week, and late respondents, who participated after receiving a reminder message. Furthermore, to account for potential common method bias (CMB), both Harman’s single factor test and common latent factor analysis (CLF) were applied as precautionary measures to validate the integrity of the findings (48).

The process of refining the scale commenced with an evaluation of the Cronbach’s alpha coefficient and the item-to-total correlation to ascertain the reliability and internal consistency of the hospital supply chain resilience scale. Subsequently, exploratory factor analysis (EFA) was employed, utilizing SPSS version 28.0, to rigorously assess the suitability of the items included. Principal component analysis (PCA) with Varimax rotation was performed to determine the Kaiser-Meyer-Olkin (KMO) measure, the communality of each item, factor loadings, and the potential for cross-loading of individual items (25). Additionally, to mitigate the risk of multi-collinearity, Variance Inflation Factors (VIF) were examined (36).

Following the EFA, confirmatory factor analysis (CFA) was conducted using the Maximum Likelihood Robustness (MLR) estimation method within the Mplus 8.3 software (49). This phase was critical for validating the initial factor structure, comprising 26 items across five dimensions, as identified by the EFA. The evaluation focused on the adequacy of parameter estimates and the model-fit indices to ensure the robustness of the overall measurement model.

Lastly, following the recommendation of previous literature (24, 25, 50), this study implemented a competing model strategy to identify the best fitting and parsimonious model for scale development. There were five alternative models being proposed. Model 1 is a null model in which all items were uncorrelated with each other. Model 2 is the model with all items loaded onto one first-order factor of supply chain resilience. Model 3 and Model 4 represent all items were loading onto a five dimensional first-order factors. The difference of them is either these five factors are uncorrelated (Model 3) or correlated (Model 4). Model 5 indicates that five dimensions of resilience capability were loaded onto the second-order factor of supply chain resilience.

## 4 Results

### 4.1 Demographic information

The selected hospitals in our sample represent about 60% of all secondary-level and above institutions across the 16 cities within Province S. As indicated in Table 4, these sample hospitals display a relatively balanced distribution across various key characteristics, including hospital level, hospital type, supply chain type, and operational model. This balanced representation allows the sample to accurately mirror the general state of hospital supply chain management across China.

**Table 4:** Demographic information of samples (*N* = 387).

	Number	%
Hospital Level		
Tertiary hospital	170	43.93
Secondary hospital	217	56.07
Hospital Type		
General hospital	220	56.85
Traditional Chinese medicine hospital	53	13.70
Speciality hospital	114	29.45
Supply Chain Type		
Medical consumable supply chain	161	41.60
Pharmaceutical products supply chain	226	58.40
Supply Chain Operation Model		
Hospital self-managed supply chain	251	64.86
Outsourced supply chain	136	35.14

### 4.2 Bias control and assessment

The analysis showed no significant differences between early and late response groups. Furthermore, Harman’s single-factor test indicated that the eigenvalues for the five factors were all greater than 1, with the first factor accounting for 48.690% of the variance, which is less than 50%, preliminarily suggesting that CMB is not a significant concern in this study. Regarding the CLF, the addition of a common latent factor to the five-factor structural equation model did not result in a significant change in the model’s chi-square value (Δ*χ*2 = 35.346, Δdf = 1), and the changes in the main fit indices including CFI (Comparative Fit Index), TLI (Tucker-Lewis Index), SRMR (Standardized Root Mean Square Residual) and RMSEA (Root Mean Square Error of Approximation) all ranged between 0.002 and 0.029, which are not substantial. Therefore, it can be inferred that CMB does not significantly affect the dataset’s results.

### 4.3 Reliability assessment

The scale refinement begins with an item analysis, utilizing critical ratio values and item-total correlation as metrics to assess the content of the questionnaire. The results of the item analysis (shown in Table 5) reveal that all items have t-values greater than 3 and Pearson product–moment correlation coefficients greater than 0.4, indicating that the scale items possess strong discriminative power and exhibit good homogeneity among the items.

**Table 5:** Item analysis results (*N* = 387).

Item	Extreme Comparison		Item-to-total correlation	
	*T* value	*p* value	Pearson’s *r*	*p* value
ANT1	13.819	<0.001	0.617	<0.001
ANT2	14.094	<0.001	0.635	<0.001
ANT3	21.786	<0.001	0.699	<0.001
ANT4	11.181	<0.001	0.545	<0.001
ANT5	16.019	<0.001	0.686	<0.001
ANT6	13.277	<0.001	0.632	<0.001
ADA1	15.033	<0.001	0.698	<0.001
ADA2	18.558	<0.001	0.769	<0.001
ADA3	15.719	<0.001	0.754	<0.001
ADA4	13.794	<0.001	0.709	<0.001
ADA5	16.270	<0.001	0.752	<0.001
ADA6	15.291	<0.001	0.689	<0.001
RES1	20.565	<0.001	0.737	<0.001
RES2	16.326	<0.001	0.689	<0.001
RES3	20.798	<0.001	0.762	<0.001
RES4	17.415	<0.001	0.730	<0.001
RES5	20.105	<0.001	0.762	<0.001
REC1	14.797	<0.001	0.673	<0.001
REC2	15.719	<0.001	0.727	<0.001
REC3	13.255	<0.001	0.681	<0.001
REC4	14.073	<0.001	0.624	<0.001
LEA1	14.852	<0.001	0.718	<0.001
LEA2	17.126	<0.001	0.736	<0.001
LEA3	16.416	<0.001	0.635	<0.001
LEA4	15.880	<0.001	0.663	<0.001
LEA5	15.170	<0.001	0.724	<0.001

ANT, Anticipation; ADA, Adaption; RES, Response; REC, Recovery; LEA, Learning.

The Kaiser-Meyer-Olkin (KMO) statistic stands at 0.940, and the Bartlett’s test of sphericity yields a *p*-value less than 0.001, suggesting that the scale items are highly suitable for factor analysis. The communalities of the individual items range from 0.613 to 0.878, meeting the retention criteria. After performing Varimax rotation to extract five factors (outlined in Table 6), the results exhibit a total cumulative variance contribution of 77.307%, which satisfies the requirement of unidimensionality.

**Table 6:** Rotated component matrix.

Item	Components				
	1	2	3	4	5
ANT1	0.237	0.071	0.087	**0.776**	0.191
ANT2	0.007	0.256	0.133	**0.753**	0.280
ANT3	0.340	0.210	0.304	**0.622**	0.025
ANT4	0.062	0.133	0.094	**0.767**	0.130
ANT5	0.331	0.131	0.306	**0.692**	0.020
ANT6	0.087	0.177	0.122	**0.855**	0.149
ADA1	0.176	**0.866**	0.193	0.160	0.108
ADA2	0.249	**0.775**	0.282	0.190	0.185
ADA3	0.173	**0.744**	0.225	0.220	0.326
ADA4	0.170	**0.732**	0.287	0.123	0.268
ADA5	0.281	**0.691**	0.314	0.183	0.178
ADA6	0.259	**0.701**	0.122	0.209	0.211
RES1	**0.843**	0.256	0.176	0.202	0.085
RES2	**0.892**	0.177	0.137	0.098	0.173
RES3	**0.825**	0.224	0.263	0.193	0.134
RES4	**0.835**	0.201	0.164	0.154	0.232
RES5	**0.798**	0.223	0.208	0.194	0.236
REC1	0.366	0.290	0.097	0.110	**0.739**
REC2	0.179	0.287	0.290	0.202	**0.799**
REC3	0.180	0.244	0.241	0.191	**0.805**
REC4	0.139	0.201	0.203	0.298	**0.650**
LEA1	0.199	0.254	**0.777**	0.194	0.214
LEA2	0.255	0.223	**0.776**	0.161	0.271
LEA3	0.238	0.151	**0.798**	0.162	0.067
LEA4	0.112	0.259	**0.802**	0.199	0.127
LEA5	0.146	0.330	**0.763**	0.155	0.266

ANT, Anticipation; ADA, Adaption; RES, Response; REC, Recovery; LEA, Learning.

According to Table 7, within the extracted five factors, Factor 1 includes five items representing the dimension of response; Factor 2 comprises six items pertaining to the adaptation dimension; Factor 3 contains five items related to the learning dimension; Factor 4 is made up of six items that correspond to the anticipation dimension; and Factor 5 consists of four items that define the recovery capability dimension. The loadings of the items range from 0.622 to 0.892, all surpassing the threshold of 0.5, indicating strong inter-dimension correlations and the absence of cross-loading issues. The Variance Inflation Factors (VIF) for all constructs are less than 5, which signifies that multicollinearity was not a concern for our study. To evaluate the reliability of the measurement construct, both Cronbach’s alpha coefficient and CR (Composite Reliability) values were employed. The Cronbach’s alpha values for the individual dimensions ranged from 0.892 to 0.956, with the overall scale achieving a Cronbach’s alpha coefficient of 0.940. The CR values were all above 0.7, indicating good internal consistency and high overall reliability of the scale.

**Table 7:** Exploratory factor analysis.

	Response	Adaption	Learning	Anticipation	Recovery
Reliability	0.956	0.932	0.927	0.895	0.892
Communality	0.824–0.856	0.662–0.855	0.747–0.808	0.635–0.807	0.613–0.878
Factor loading	0.798–0.892	0.691–0.866	0.763–0.802	0.622–0.855	0.650–0.805
Eigen value	12.625	2.265	2.144	1.677	1.354
Cumulative variance (%)	48.69%	57.40%	62.65%	72.10%	77.31%

KMO = 0.94, Total variance extracted = 77.31%, Number of factors extracted = 5.

### 4.4 Validity assessment

Content validity was ensured throughout the scale development process, which included item modification and validation based on literature review, Delphi studies, and expert panel reviews.

The AVE (Average variance extracted), CR values, and factor loadings for the five dimensions are presented in Table 7. The factor loadings ranged from 0.680 to 0.943, all exceeding the threshold of 0.5 and statistically significant. The CR and AVE values varied from 0.899 to 0.956 and from 0.599 to 0.813, respectively. With all dimension AVE values exceeding 0.5 and CR values above 0.7, the scale exhibits good convergent validity.

Discriminant validity was assessed by comparing the square root of the AVE with the correlation estimates between constructs. As indicated in Table 8, the correlation coefficients between dimensions were all lower than the square root of the respective AVE values, suggesting good discriminant validity of the scale.

**Table 8:** Measure of model validity.

	Cronbach’s α	AVE	CR	ANT	ADA	RES	REC	LEA
ANT	0.895	0.599	0.899	0.774				
ADA	0.932	0.701	0.933	0.550**	0.837			
RES	0.956	0.813	0.956	0.496**	0.597**	0.902		
REC	0.892	0.703	0.903	0.537**	0.673**	0.544**	0.838	
LEA	0.927	0.723	0.928	0.533**	0.679**	0.554**	0.634**	0.850

ANT, Anticipation; ADA, Adaption; RES, Response; REC, Recovery; LEA, Learning. Diagonal = square root of AVE; ** means significance of correlations at *p* < 0.001.

### 4.5 Assessment of competing model

The model fit indices of five competing model were illustrated in Table 9. In general, a model with a χ2/df less than 3, CFI and TLI greater than 0.9, SRMR and RMSEA less than 0.08, and a low BIC (Bayesian Information Criterion) value is considered to have a good fit. According to these results, Model 1, Model 2 and Model 3 are unacceptable, since all values of model fit indices were not satisfied with minimum threshold criteria. Both Model 4 and Model 5 achieved satisfactory model fit, while Model 5 demonstrated slightly better model fit statistically. Model 5, which emerged as the superior model, posits that the five dimensions of resilience capability—anticipation, adaptation, response, recovery, and learning—are not merely independent constructs but are interlinked and contribute to a higher-order factor of supply chain resilience. This finding supports the dynamic capability perspective, which advocates for an integrated approach to resilience, emphasizing the need for hospitals to possess multi-dimensional capabilities.

**Table 9:** Model fit indices of alternative model.

Model	*χ*^2^	*df*	*χ*^2^/*df*	CFI	TLI	BIC	SRMR	RMSEA
Model 1	6710.773	325	20.649			35486.604	0.447	0.225
Model 2	3086.664	299	10.323	0.563	0.525	30401.299	0.111	0.155
Model 3	1040.630	293	3.552	0.883	0.870	27498.241	0.234	0.081
Model 4	839.170	289	2.904	0.914	0.903	27255.794	0.057	0.07
Model 5	839.961	294	2.85	0.915	0.905	27227.574	0.058	0.069

## 5 Discussion

### 5.1 Interpretation of constructs

In the context of healthcare institutions, which represent the downstream echelon of the healthcare supply chain, the constructs retained in this study exhibit notable deviations from the extant literature that focuses on the entire supply chain or the upstream manufacturing sector. In this study, dynamic capabilities manifest through the ability to foresee potential disruptions (anticipation), modify operations and processes in response to changes in the environment (adaption), react promptly to immediate threats (response), restore normal operations efficiently after a disruption (recovery), and learn from past experiences to improve future performance (learning).

The first construct, anticipation, concentrates on the capability to identify and anticipate potential risks, echoing the proactive strategies commonly espoused by existing literature (9, 19). This study has illustrated six pivotal items that constitute anticipation: enhancing awareness of potential vulnerabilities, bolstering demand forecasting, forging strategic relationships with key suppliers, augmenting the robustness of the supply chain network, improving information transparency, and cultivating a culture and training framework for pre-disruption risk management. While prior research suggests that manufacturers possess the proactive capability to dynamically reconfigure supply chain network, including location, density, complexity, and even product design (51–53), hospitals, by contrast, are constrained to monitoring the robustness and security of their supply chain networks preemptively, without the ability to determine these factors.

The second construct, adaption, encompasses the enhancement of flexibility and the preservation of redundancy. Scholars have previously illuminated the advantages of bolstering flexibility within the manufacturing supply chain, with reference to flexible processes (37), order fulfillment (7), and transportation routes (54). Notably, this study introduces an additional item pertaining to institutional flexibility, reflecting the unique context of Chinese public hospitals where institutional considerations often take precedence over processes. Regarding redundancy, this study retains the two items—inventory redundancy (7, 38) and facility redundancy (55)—highlighted in earlier research. Additionally, an item addressing personnel redundancy has been incorporated, likely in response to the acute staffing challenges faced by hospitals during the COVID-19 period.

The third construct, response, is predicated on the premise of supply chain collaboration with key partners and the agility to respond swiftly to disruptions. The collaborative items encompass both internal cross-functional departmental planning within the hospital (horizontal) and external coordination with key suppliers (vertical), as well as real-time information sharing to ensure an immediate response. These elements align with previous literature from other industries (7, 16, 17). Agility in this study is characterized by a convenient risk information feedback system, expedited reactions to disruptive events, and a comprehensive emergency plan and team. The inclusion of the emergency plan and team within this dimension underscores the need for timely and efficient disruption response, although it has also been considered a part of reactive contingency plans for post-disruption recovery in prior literature (19). Given this study’s focus on immediate risk response, situating it within the ‘response’ dimension is deemed appropriate.

The fourth construct, recovery, involves the reconfiguration of resources, the efficiency of recovery operations, and government support. The capability to restructure the supply chain and redeploy various resources aligns with findings in existing literature (8, 39, 51). Notably, the item reflecting the financial strength of an organization, prevalent in previous studies (34), was excluded during the Delphi process. This exclusion may be attributed to the fact that the healthcare institutions surveyed are non-profit public hospitals, whose financial robustness largely depends on government backing. Hence, conventional metrics for assessing market position may not translate effectively to the public hospital context.

The final construct, learning, concentrates on post-disruption knowledge management and the enhancement of social capital for mutual learning. Prior research has shown that both intentional and unintentional learning can positively influence resilience-building (56), particularly with unintentional learning potentially fostering greater employee engagement. However, this study exclusively selected items pertaining to intentional learning, such as post-disruption feedback, post-disruption training and education, and the application of innovative technologies in educational initiatives. The latter two items emphasize leveraging social capital by nurturing inter-organizational relationships and trust with supply chain stakeholders. This collaborative learning approach enables hospitals to transcend organizational boundaries and engage in co-creation processes that amplify learning capabilities (26, 47), preparing them more robustly for future challenges. It is particularly noteworthy that the capacity for learning from disruptions, despite its significance, remains an underexplored domain within the literature (19, 56). Therefore, this study responds to the call on exploring the learning effect on supply chain resilience and expand the breadth of measuring supply chain resilience.

### 5.2 Practical implications

The hospital supply chain resilience scale developed through this research provides hospital managers with a robust instrument to assess and enhance their supply chain’s resilience. This tool can be employed periodically to monitor changes across various dimensions of resilience, allowing managers to track progress and make informed decisions to fortify their supply chains against potential disruptions and identify specific areas that require attention and improvement. In addition, the scale enables hospitals to benchmark their resilience levels with those of their peers. This comparison can reveal best practices and innovative strategies that other institutions have successfully implemented. Hospital managers can learn from these exemplars and adapt relevant practices to their own contexts. Moreover, the scale can facilitate communication and collaboration among supply chain stakeholders. By using a common language and framework to discuss resilience, hospital managers can more effectively engage with suppliers, distributors, and other partners to co-create resilience strategies. The scale can serve as a foundation for joint problem-solving and continuous improvement efforts. Furthermore, the scale can also inform policymaking and resource allocation at the regional or national level. Health authorities can use the scale to assess the overall resilience of the healthcare supply chain and identify systemic vulnerabilities that require intervention. This information can guide targeted investments in infrastructure, technology, or capacity-building initiatives to enhance the resilience of the entire healthcare system.

### 5.3 Limitations and future directions

While this study has made significant strides in understanding hospital supply chain resilience, it is not without its limitations, which in turn open avenues for future research opportunities. One limitation of this study is its geographical concentration on healthcare institutions within mainland China. The unique healthcare policies, market dynamics, and cultural factors could potentially limit the applicability of the proposed multi-dimensional resilience measurement scale in different contexts. However, it’s important to note that the selection of hospitals in this study was carefully considered to include a broad spectrum of institutions, encompassing urban and rural settings, large and small hospitals, general and specialized facilities, as well as a variety of hospital supply chain operational models and levels of supply chain complexity. This diverse sample helps to represent the current situation of various medical institutions more accurately, thereby partially mitigating the mentioned limitation. To further enhance the generalizability and applicability of the resilience measurement scale, future research should expand its scope by including a wider array of geographical locations and healthcare contexts to validate the robustness of the scale across different settings. Additionally, the study’s measurement scale, while validated, could be refined to include emerging threats and novel resilience strategies that continue to evolve with the rapidly changing landscape of global health emergencies. As such, continuous updates and validations of the scale are necessary. Future research could also focus on developing metrics to assess the learning processes within hospital supply chains and how these contribute to long-term resilience and systemic improvements.

## 6 Conclusion

This study embarked on an extensive literature review, enriched by expert interviews and Delphi studies, to pinpoint a robust set of 26 items designed to measure the resilience of hospital supply chains. Advancing to the empirical phase, the study disseminated online surveys across a broad spectrum of 387 healthcare institutions in Province S of mainland China. These items encapsulate the core dimensions of anticipation, adaptation, response, recovery, and learning—each a critical facet of resilience as viewed through the lens of dynamic capability. The rigorous evaluation confirmed the reliability and validity of the measurement scale, underscoring its effectiveness in capturing the complex nature of hospital supply chain resilience.

## Data availability statement

The raw data supporting the conclusions of this article will be made available by the authors, without undue reservation.

## Author contributions

BD: Conceptualization, Formal analysis, Funding acquisition, Methodology, Project administration, Resources, Validation, Writing – original draft, Writing – review & editing. XY: Data curation, Formal analysis, Investigation, Methodology, Writing – original draft. TG: Data curation, Resources, Writing – review & editing. ZL: Data curation, Investigation, Writing – review & editing. QS: Conceptualization, Supervision, Writing – review & editing.
